# Molecular characterization and heterologous expression of two α-glucosidases from *Metschnikowia* spp, both producers of honey sugars

**DOI:** 10.1186/s12934-020-01397-y

**Published:** 2020-07-11

**Authors:** Martin Garcia-Gonzalez, Marina Minguet-Lobato, Francisco J. Plou, Maria Fernandez-Lobato

**Affiliations:** 1 Department of Molecular Biology, Centre for Molecular Biology Severo Ochoa (CSIC-UAM), University Autonomous from Madrid. C/Nicolás Cabrera, 1. Cantoblanco, 28049 Madrid, Spain; 2 Institute of Catalysis and Petrochemistry, CSIC, C/Marie Curie, 2. Cantoblanco, 28049 Madrid, Spain

**Keywords:** *Metschnikowia*, α-glucosidase, Transglucosylation, GH13, Hetero-gluco-oligosaccharides, Isomelezitose, Trehalulose, Esculose

## Abstract

**Background:**

α-Glucosidases are widely distributed enzymes with a varied substrate specificity that are traditionally used in biotechnological industries based on oligo- and polysaccharides as starting materials. According to amino acid sequence homology, α-glucosidases are included into two major families, GH13 and GH31. The members of family GH13 contain several α-glucosidases with confirmed hydrolytic activity on sucrose. Previously, a sucrose splitting activity from the nectar colonizing yeast *Metschnikowia reukaufii* which produced rare sugars with α-(1→1), α-(1→3) and α-(1→6) glycosidic linkages from sucrose was described.

**Results:**

In this study, genes codifying for α-glucosidases from the nectaries yeast *M. gruessii* and *M. reukaufii* were characterised and heterologously expressed in *Escherichia coli* for the first time. Recombinant proteins (Mg-αGlu and Mr-αGlu) were purified and biochemically analysed. Both enzymes mainly displayed hydrolytic activity towards sucrose, maltose and *p*-nitrophenyl-α-d-glucopyranoside. Structural analysis of these proteins allowed the identification of common features from the α-amylase family, in particular from glycoside hydrolases that belong to family GH13. The three acidic residues comprising the catalytic triad were identified and their relevance for the protein hydrolytic mechanism confirmed by site-directed mutagenesis. Recombinant enzymes produced oligosaccharides naturally present in honey employing sucrose as initial substrate and gave rise to mixtures with the same products profile (isomelezitose, trehalulose, erlose, melezitose, theanderose and esculose) previously obtained with *M. reukaufii* cell extracts. Furthermore, the same enzymatic activity was detected with its orthologous Mg-αGlu from *M. gruessii*. Interestingly, the isomelezitose amounts obtained in reactions mediated by the recombinant proteins, ~ 170 g/L, were the highest reported so far.

**Conclusions:**

Mg/Mr-αGlu were heterologously overproduced and their biochemical and structural characteristics analysed. The recombinant α-glucosidases displayed excellent properties in terms of mild reaction conditions, in addition to pH and thermal stability. Besides, the enzymes produced a rare mixture of hetero-gluco-oligosaccharides by transglucosylation, mainly isomelezitose and trehalulose. These compounds are natural constituents of honey which purification from this natural source is quite unviable, what make these enzymes very interesting for the biotechnological industry. Finally, it should be remarked that these sugars have potential applications as food additives due to their suitable sweetness, viscosity and humectant capacity.

## Background

The already proven relationship between food and health has promoted a growing biotechnological interest in functional foods and nutraceutical ingredient markets, where bioactive oligosaccharides are gaining relevance among compounds such as dietary fibres, peptides, polyols or unsaturated fatty acids [1–3]. Bioactive oligosaccharides include fructo-oligosaccharides (FOS), galacto-oligosaccharides (GOS), gluco-oligosaccharides (GlcOS), chito-oligosaccharides (COS) and xylo-oligosaccharides (XOS) among others [4, 5]. In GlcOS, α-glucose units can be connected by α-(1→6), α-(1→4), α-(1→3) or α-(1→2) links giving rise to isomalto-oligosaccharides (IMOs), malto-oligosaccharides (MOS), nigero-oligosaccharides and koji-oligosaccharides, respectively [6]. In addition, in GlcOS glucose monomers can also be linked to a non-glucosidic unit generating hetero-GlcOS as occurs with erlose, leucrose, melezitose, thenaderose or trehalulose, carbohydrates found in natural sources like honey or sugar cane [7–9].

Several bioactive oligosaccharides including FOS (inlulin and ^1^F-FOS serie), GOS, GlcOS (basically IMOS), lactulose, lactosucrose and raffinose have been recognized as prebiotics agents promoting the growth of beneficial bacteria, mainly *Bifidobacterium* spp. and *Lactobacillus* spp. [10, 11]. Besides, the consumption of these sugars has also been related to a reduction in the risk of suffering colon cancer and cardiovascular diseases, or with the improvement of mineral absorption and immune system response. [12, 13]. Due to their techno-functional properties, bioactive oligosaccharides have applications in food technology as bulking and moisture retaining agents, fat and sugar substitutes, textural enhancers, and non-cariogenic ingredients, which give them great biotechnological utility [14, 15].

α-Glucosidases (EC 3.2.1.20) are glycoside hydrolases widely distributed in microorganisms, animals and plants, which catalyse the splitting of α-glucose units from the non-reducing end of different size substrates, including oligo-, polysaccharides and alkyl-α-glycosides, among others [16, 17]. According to their substrate specificity, α-glucosidases are traditionally classified into three groups. Group I includes the ones that hydrolyse heterogeneous substrates (e.g. sucrose and aryl-glucosides) more efficiently than homogeneous substrates as maltose, the group II are more active on homogeneous substrates as maltose and isomaltose and finally, the group III comprise enzymes that exhibit the same activity as the type II, but also hydrolyse long-chain substrates [16, 18]. Based on a structural classification, α-glucosidases are distributed into five glycoside hydrolase (GH) families: GH4, GH13, GH31, GH63 and GH97 [19]. The family GH13 groups more than 85 0000 proteins (http://www.cazy.org) active on substrates containing α-glucosidic linkages, and include α-amylases, cyclodextrin glucanotransferases, pullulanases, α-glucosidases and oligo-1,6-glucosidases [20, 21]. Together with families GH70 and GH77, GH13 comprise the clan GH-H, also known as the α-amylase family due to its most studied member, the *Aspergillus oryzae* α-amylase [21]. Proteins into this family show a characteristic catalytic (β/α)_8_-barrel domain, where 4–7 short conserved regions (CSR) contain key amino acids involved in the enzyme hydrolytic mechanism and a catalytic triad formed of three acid residues [22, 23].

Several α-glucosidases display transglucosylation activity and therefore, are capable of synthesising oligosaccharides and/or glyco-conjugates [24–26]. For instance, the α-glucosidase from the yeast *Saccharomyces cerevisiae* produced isomelezitose in overload sucrose reactions [27]. In addition, the α-glucosidases from *Bacillus* sp. mainly synthesised theanderose in a mixture containing isomelezitose and 4-O^F^-glucosylsucrose, and that from *B. licheniformis* produced theanderose, unless mellibiose was added to sucrose, in which the hetero-GlcOS Glc-(1→6)-Gal-(1→6)-Glc was also formed [16, 28]. In this context, the α-glucosidase from the basidiomycetous yeast *Xanthophyllomyces dendrorhous* synthesised panose, maltotriose (both major products) from maltose and an unusual tetrasaccharides mixture including α-(1→6) links [17].

*Metschnikowia reukaufii* and *M. gruessii* (*Ascomycota, Saccharomycetales*) are ubiquitous budding yeasts that colonise floral nectaries [29, 30]. These microorganisms grow in high sugar content environments (~ 40% w/v) and presumably could contribute to the final composition of the nectar [31], which a priori would make them interesting sources for novel enzymatic activities related with sucrose transformation. In fact, in a previous work, we have detected a sucrose splitting activity in *M. reukaufii* cell extracts that produced hetero-GlcOS naturally present in honey, mainly isomelezitose and trehalulose [32]. Here, we have isolated, characterised and analysed the gene responsible for the aforementioned activity in both yeast species. Besides, a 3D model of these proteins based on the *S. cerevisiae* oligo-1,6-glucosidase were proposed and several structural features, typical of the family GH13, were identified.

## Results and discussion

### Isolation and heterologous expression of α-glucosidases from two *Metschnikowia* species

As mentioned before, we have recently reported a potential α-glucosidase activity in *M. reukaufii* cell extracts that was able to hydrolyse sucrose and produced, by transglucosylation, different honey oligosaccharides, mainly isomelezitose and trehalulose [32]. Genomic DNA from *M. reukaufii* strain MR1 was previously sequenced and *de novo* assembled, which allowed Drs. M.K. Dhami and T. Fukami, locate a putative oligo-1,6-glucosidase coding sequence [30]. This sequence (ID CM010598.1) aligned with part of potential α-glucosidases from ascomycetous yeasts such as *M. biscupidata* (XP018711500.1, query coverage 87%, identity 86.4%), *Candida intermedia* (SGZ57444.1, query coverage 87%, identity 85.7%) and *Clavispora lusitaniae* (OVF11230, query coverage 85%, identity 85.6%). In order to isolate the gene which encodes the α-glucosidase that could be responsible for the activity previously detected in *M. reukaufii* cell extracts, oligonucleotides directed to the ID CM010598.1 sequence ends were designed and used in PCR reactions containing genomic DNA from *M. gruessii* and *M. reukaufii*. In both cases, a DNA fragment of ~ 1700 bp was amplified, whose theoretical translation gave rise to a protein of 566-amino acids showing high similarities with glucosidases included in the glycoside hydrolase family GH13. Best homology results were obtained against the oligo-1,6-glucosidase from *Saccharomyces cerevisiae* (NP011803.3, query coverage 98%, identity 49.2%), with proved hydrolytic activity on isomaltose and maltose, as well as α-glucosidases from *Schizosaccharomyces pombe* (NP595063.1, query coverage 97%, identity 46.3%) and *Bacillus subtilis* (NP391336.1, query cover 97%, identity 44.0%), both able to hydrolyse pNPG, maltose and sucrose [33–35].

**Fig. 1:**
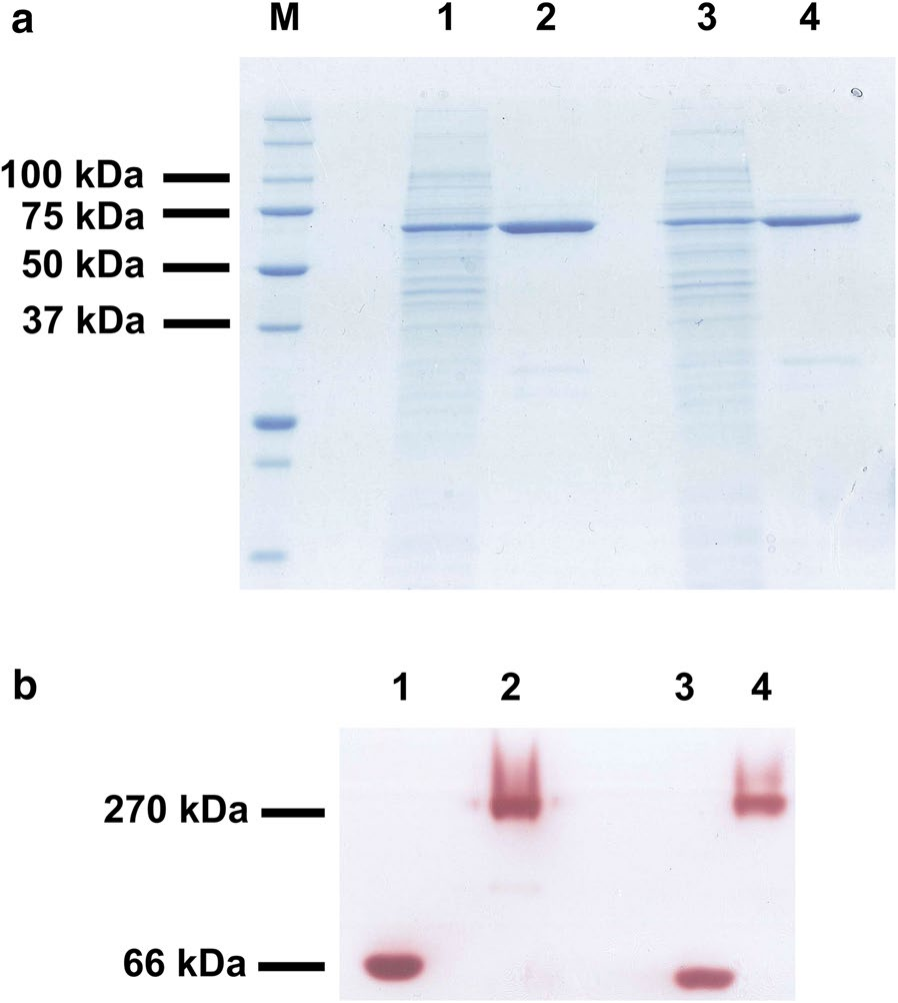
SDS-PAGE analysis of the α-glucosidases from *Metsnikowia* species expressed in *E. coli*. Non-precipitated cell lysates of the bacteria including the constructions Mg-αGLU-pET28b(+) (*lane 1*) and Mr-αGLU-pET28b(+) (*lane 3*) were loaded. Ni-Sepharose purified fractions containing recombinant proteins Mg-αGlu (*lane 2*) and Mr-αGlu (*lane 4*) were also evaluated (**a**). Zymogram analysis using 1 M sucrose as substrate and purified Mg-αGlu (*lane* 1) or Mr-αGlu (*lane 3*). Commercial *S. cerevisiae* invertase was used as positive control (*lanes 2 and 4*) (**b**). Proteins in the range of 1–3 μg were loaded in each well. Numbers at the left of panels (**a**) and (**b**) indicate the positions of the molecular mass standards in kDa

Sequences of Mg/Mr-αGlu were practically identical to each other showing a 99.3% identity on a 100% coverage with a theoretical molecular weight of 65,763 and 65811 Da, respectively. The two open reading frames (ORFs) encoding the potential α-glucosidases from *M. gruessii* and *M. reukaufii*, were included in constructions MgαGLU-pET28b(+) and MrαGLU-pET28b(+), and then transformed in *Escherichia coli*. The two recombinant proteins were successfully expressed in this bacterium because a major protein of ~ 66 kDa was detected by SDS-PAGE in the soluble fractions (Fig. 1a). Proteins were purified (Additional file 1: Table S1) and their hydrolytic activity was proved by zymogram analysis (Fig. 1b) as referred in the “Methods” section.

### Amino acid sequence analysis and identification of the catalytic residues

**Fig. 2:**
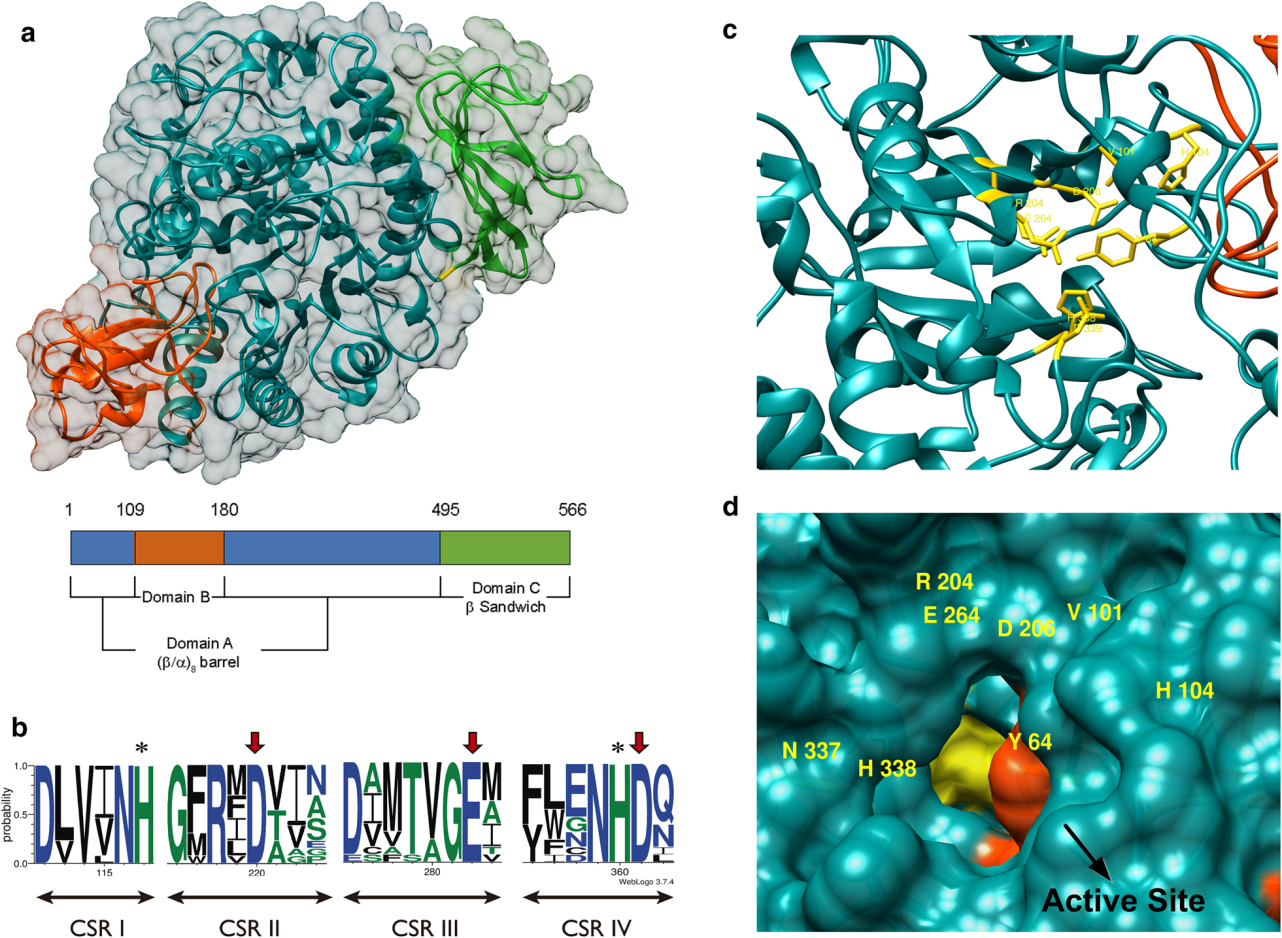
Preliminary structural analysis of recombinant α-glucosidases. Superposition of the overall structures of Mg/Mr-αGlu based on the *S. cerevisiae* oligo-1,6-glucosidase (PDB: 3A47_A) template. The tree typical domains of the glycoside hydrolase family GH13 were depicted using the colour code given in the scheme (**a**). Sequence logo of the four conserved regions from the family GH13 identified in Mg/Mr-αGlu proteins. Amino acids of the catalytic triad were indicated with red arrows. The rest of highly conserved residues important for the catalytic mechanism were marked with asterisks. The logo was performed using 11 characterised microbial glycoside hydrolases of the family GH13 (PDB: 3A47_A; 1UOK_A; 5DO8_A; 4MB1_A; 5BRQ_A; 5ZCB_A; 2ZE0_A; 4AIE_A; 1M53_A; 1ZJA_A and UniProtKB: Q9P6J3) (**b**). Schematic view of the conserved residues (shown in yellow) of Mg/Mr-αGlu potentially implicated in subsite -1: Y64, V101, H104, R204, D206, E264, H338 and D339 (**c**). Surface representation of the active site entrance. Catalytic domain was coloured in dark cyan and domain B in red orange. The subsite -1 surface was highlighted in yellow and residues implicated were labelled (**d**)

Overall deduced sequences of the two proteins from *Metschnikowia* species were very similar to 3D-structurally characterized proteins such as oligo-1,6-glucosidases from *S. cerevisiae* and *B. cereus*, and α-glucosidases from *S. pombe* and *B. subtilis*. In this work, the structural models of Mg/Mr-αGlu were obtained based on the *S. cerevisiae* oligo-1,6-glucosidase (Fig. 2a), which gave the best sequence homology results. The three domains A, B and C characteristic of the α-amylase family (Clan GH-H) were identified in both proteins [20, 23]. The domain A (catalytic domain) adopted the typical (β/α)_8_-barrel fold and was comprised of eight inner β-strands surrounded by eight α-helices (Additional file 1: Fig. S1). Domain B constituted a large loop protruding from the (β/α)_8_-barrel connecting the β-strand 3 and the α-helix 3. This domain is variable among enzymes included in the α-amylase family and has been proposed that, together with domain A, contained amino acids involved in enzyme specificity. A C-terminal antiparallel β-sandwich domain (Domain C) could be related to the catalytic domain stabilization and substrate binding [21, 22].

The structural analysis of the two α-glucosidases from *Metschnikowia* species was performed by multiple alignment (Additional file 1: Fig. S1) and sequence logo (Fig. 2b). The comparison of Mg-αGlu and Mr-αGlu sequences was done using eleven structurally characterised glycoside hydrolases from family GH13 (CAZy database) with proved function. Both α-glucosidases here analysed harbour the four conserved sequence regions (CSR) I-IV characteristic of family GH13 members with several highly conserved residues, including the three acid groups potentially implicated in the hydrolytic mechanism (catalytic triad). Thus, Asp206 and Glu264 would act as the catalytic nucleophile and proton donor, respectively, in the glycoside bond cleavage, whilst Asp339 could be involved in stabilising the transition state [21]. The relevance of these three amino acids for the hydrolytic activity was confirmed by site-directed mutagenesis. Variants D206A, E264Q and D339A of Mg/Mr-αGlu were obtained, expressed in *E. coli*, and purified using the protocol already employed with the wild-type proteins. Then, mutated proteins were analysed by SDS-PAGE and the hydrolytic activity was evaluated by both, zymograms and DNS assays. Contrarily to the wild-type variants and the *S. cerevisiae* invertase used as positive controls, all the generated mutants (D206A, E264Q and D339A) did not showed any activity towards sucrose. Therefore, each of these residues were essential for the hydrolytic activity of the two α-glucosidases from *Metschnikowia* yeasts (Additional file 1: Fig. S2). Moreover, other highly conserved amino acids such as Tyr64, Val101, His104, Arg204 and His338 were also identified in the potential structure of Mg/Mr-αGlu. These residues, together with the catalytic triad, were all located near or at the end of the β-strands 2, 3, 4, 5 and 7 of the (β/α)_8_-barrel (Additional file 1: Fig. S1). In fact, these amino acids are in optimal position into the active site to comprise the subsite − 1 (glycosidic bond cleavage occurs between subsites -1 and + 1) where an α-glucose moiety of the substrate to be hydrolysed is linked and temporally retained during the catalysis (Fig. 2c, d). These structural features have been widely described for glycoside hydrolases included in the family GH13 [21, 36, 37].

### Biochemical characterization of Mg-αGlu and Mr-αGlu expressed in *Escherichia coli*

**Fig. 3:**
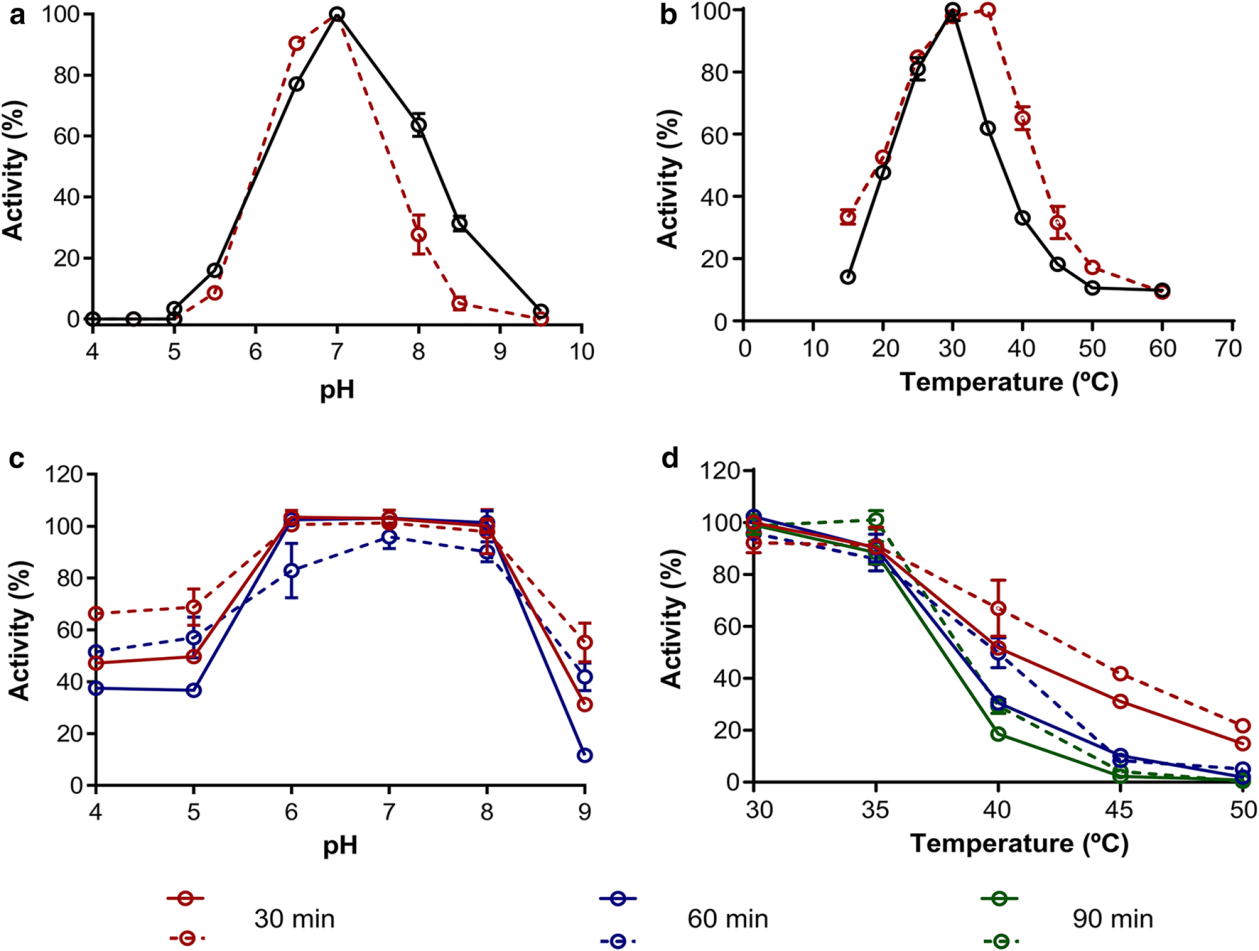
Optimal pH (**a**), optimal temperature (**b**), pH stability (**c**) and thermostability (**d**) of the purified Mg-αGlu (dotted lines) and Mr-αGlu (continuous lines). The effect of pH and temperature on the enzymatic activity was assessed on sucrose 60 g/L at 30 °C and pH 7.0, respectively. For enzyme stability profiles, the recombinant proteins were incubated for 30 min (red lines), 60 min (blue lines) and 90 min (green lines) over different pH and temperature ranges prior to the addition of the substrate. Then, remaining activity was evaluated at 30 °C and pH 7.0, as described in the “Methods” sections. Experiments were performed in triplicate and the standard errors are indicated

The sucrose hydrolytic activity of the two recombinant α-glucosidases was evaluated in order to characterise the optimal conditions for enzymatic assays. Mg/Mr-αGlu displayed the maximum activity at 25–35 and 25–30 °C, respectively, and pH 6.5–7.0 (Fig. 3a, b). These values are in accordance with other yeast α-glucosidases. For instance, the α-glucosidases from *Hansenula polymorfa* and *Candida albicans* displayed optimum pH and temperature values of 6.0–7.0 units and 37 °C [38, 39], respectively. The four isomaltases from *S. cerevisiae* showed a maximum hydrolytic activity at 7.0–8.0 units of pH and 36–46 °C [40]. Mg/Mr-αGlu were quite stable in the pH ranging from 6.0–8.0, since both enzymes retained 90–100% of the initial activity after 30 and 60 min incubation in different buffers (Fig. 3c). Besides, at pH values lower than 6.0, Mg-αGlu seemed to be more stable than Mr-αGlu based on its higher residual activity (52–70% vs. 37–50%, respectively). When Mg/Mr-αGlu were incubated without substrate in the range from 30 to 50 °C for 30–90 min and then hydrolytic activity was estimated, enzymes maintained 50% of their initial activity (T_50_) at 37–44 and 30–40 °C, respectively (Fig. 3d). Thus, Mg-αGlu enzyme seemed to be a slightly more thermostable than Mr-αGlu under the aforementioned conditions.

**Fig. 4:**
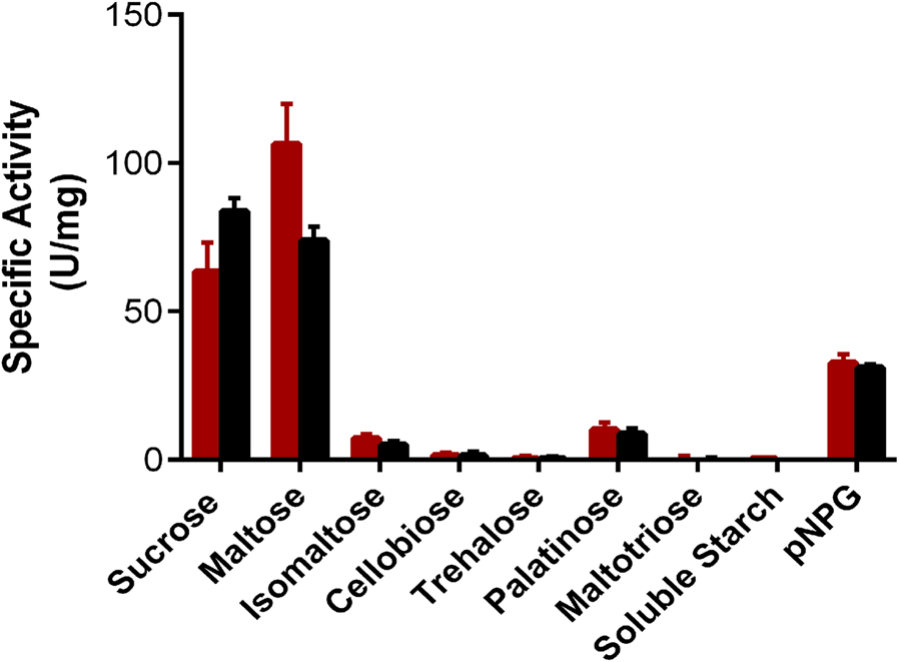
Hydrolytic activity of Mg-αGlu (*red bars*) and Mr-αGlu (*black bars*) on the referred substrates. All reactions were incubated for 20 min at 30 °C and pH 7.0. After inactivation at 80 °C, released glucose was determined using the GOD-POD colorimetric assay. Activity towards pNPG was determined by the measurement of the free pNP in the reaction. The values obtained are the average of three independent experiments. Standard errors are indicated

The hydrolytic activity of Mg/Mr-αGlu was assessed using different substrates and both enzymes preferably hydrolysed sucrose and maltose, followed by pNPG. However, Mg-αGlu exhibited an activity on maltose ~ 1.7-fold higher compared to sucrose while Mr-αGlu showed practically the same activity on both substrates. When using pNPG, the two recombinant enzymes displayed practically the same specific activity (Fig. 4). Several microbial type I α-glucosidases showed similar substrate specificities such as maltases from *C. albicans* and *H. polymorfa*, as well as α-glucosidases from *Bacillus* sp. SAM1606 and *B. subtilils*. Furthermore, Mg/Mr-αGlu displayed very low hydrolytic activity towards isomaltose and palatinose, while cellobiose and trehalose were nearly not hydrolysed (Fig. 4). Thus, these enzymes do not cleave β-(1→4) or α-(1→1) glycosidic bonds, as described for the α-glucosidases from *S. cerevisiae, C. albicans* and *B. subtilis* [28, 38, 41, 42]. In addition, none of the *Metschnikowia* enzymes hydrolysed maltotriose or soluble starch, rejecting they were type III α-glucosidases that hydrolyse long-chain substrates.

Enzyme kinetics using sucrose and maltose as substrates were examined for both recombinant enzymes (Additional file 1: Fig. S3). Analysis of the kinetic parameters showed that Mg-αGlu hydrolysed maltose with a catalytic efficiency (*k*_cat_*/K*_m_) 1.7-fold higher than Mr-αGlu. However, Mr-αGlu was 1.5-fold more efficient hydrolysing sucrose than Mg-αGlu (Table 1). Interestingly, the α-glucosidase III from the European honeybee (HBG-III), *Apis meliffera* L expressed in *Pichia pastoris*, exhibited sucrose-comparable kinetic parameters to the recombinant *M. reukaufii* α-glucosidase, with a catalytic efficiency ~ 5.3 s^−1^ mM^−1^ [26]. Due to native Mg-αGlu, Mr-αGlu and HBG-III are capable of hydrolysing sucrose at nectaries conditions, where sugars concentration reach values higher than 40%, is not surprising that these enzymes showed high *K*_m_ values for this substrate ranging from ~ 42 to ~ 65 mM. In fact, native HBG-III secreted by the hypo pharyngeal glands of bees resulted to be the α-glucosidase previously purified from honey [43].

**Table 1:** Catalytic constants of α-glucosidases from *M. gruessii* and *M. reukaufii*

Substrate	Sucrose			Maltose		
Enzyme	*K*_m_ (mM)	*k*_cat_ (s^−1^)	*k*_cat_/*K*_m_ (s^−1^mM^−1^)	*K*_m_ (mM)	*k*_cat_ (s^−1^)	*k*_cat_/*K*_m_ (s^−1^mM^−1^)
Mg-αGlu	54.1 ± 5.8	269.7 ± 10.9	4.9 ± 0.7	42.8 ± 5.6	365.6 ± 16.7	8.5 ± 1.5
Mr-αGlu	64.9 ± 4.6	477.2 ± 13.7	7.4 ± 0.7	79.8 ± 12.8	405.9 ± 28.1	5.1 ± 1.2

Reaction rates measurements were performed by triplicate. Values of kcat were calculated from Vmax considering the theoretical molecular mass of 66 kDa. Standard errors are indicated

Based on the hydrolytic profile mentioned above, the recombinant proteins here analysed could be considered as type I α-glucosidases with the capability to cleave glycosidic bonds from heterogeneous substrate like sucrose or pNPG.

### Characterization of the transglucosylation activity of recombinant α-glucosidases

In order to evaluate the potential implication of Mg/Mr-αGlu in hetero-GlcOS synthesis, reactions with 30 U of hydrolytic activity and 500 g/L sucrose were performed. Then, the sugar composition of the mixtures was analysed by HPAEC-PAD. Reactions with *M. reukaufii* enzymatic extract were also carried out under the same conditions and used as controls.

**Fig. 5:**
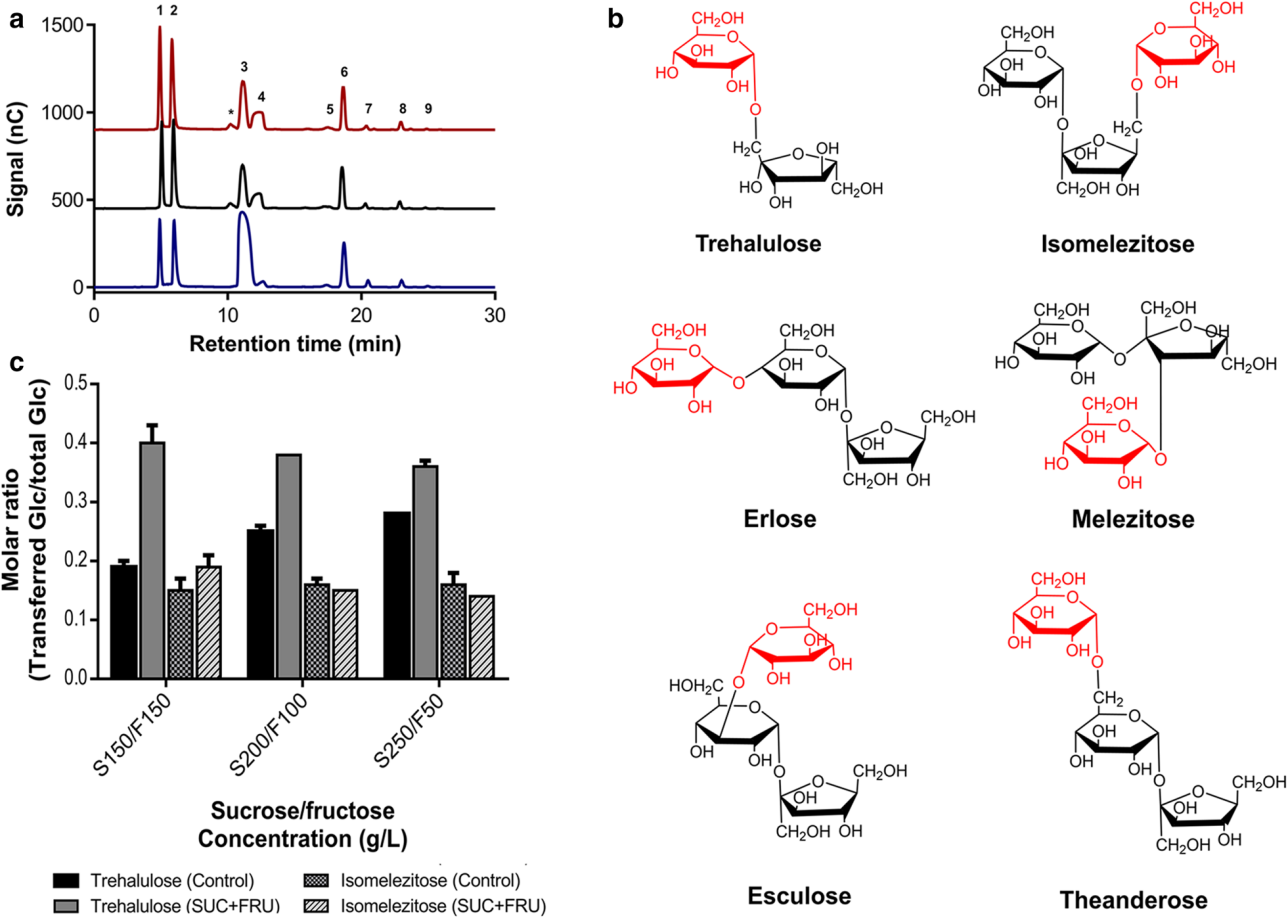
Representative HPAEC-PAD chromatogram of transglucosylation products synthesised in overload sucrose reactions. Chromatograms correspond to the sugar mixtures obtained by using *M. reukaufii* cell extract (*blue line*), Mr-αGlu (*black line*) or Mg-αGlu (*red line*) for about 71–76% sucrose conversion. Peaks assignation: (1) glucose, (2) fructose, (3) sucrose, (4) trehalulose, (5) melezitose, (6) isomelezitose, (7) theanderose, (8) erlose, (9) esculose, (*) non-identified peak (**a**). Chemical structures of the oligosaccharides synthesised by *Metschnikowia* yeasts α-glucosidases. The glucose unit transferred to free fructose or sucrose is represented in red (**b**). Molar ratio of glucose transferred to trehalulose or isomelezitose related to the total initial glucose. Reactions using different initial concentrations of sucrose plus fructose or sucrose (control reactions) were performed in triplicate. Standard errors are indicated (**c**)

Interestingly, similar chromatographic profiles were obtained in all the reaction analysed, and several peaks corresponding to transglucosylation products were identified. Differing from control reactions, when recombinant α-glucosidases were used, a new peak (non-identified) was detected, possibly a disaccharide due to its retention time (Fig. 5a). Isomelezitose and trehalulose were the main oligosaccharides produced, and to a lesser extent, the trisaccharides erlose, melezitose, theanderose and esculose. As we reported elsewhere, the glucose unit was principally transferred to the more reactive primary hydroxyl groups [25, 32]. Thus, isomelezitose and trehalulose were produced when the positions 6-OH of the sucrose β-fructofuranosyl unit and 1-OH of the free fructose were glucosylated, respectively. Melezitose, erlose, theanderose and esculose were also obtained by the glucosylation of positions 3-OH of the β-fructofuranosyl unit, 4-OH, 6-OH and 3-OH of the α-glucopyranosyl unit of sucrose, respectively (Fig. 5b and Additional file 1: Fig. S4). Despite further analysis is required, the current results may indicate that native α-glucosidase from *M. reukaufii* (present in the cell extracts) was involved in the hetero-GlcOs biosynthesis described before [32]. The production of these sorts of oligosaccharides by α-glucosidases from the family GH13 was previously described (Additional file 1: Table S2). For instance, the production of isomelezitose was firstly described by Chiba et al. employing the α-glucosidase from *S. cerevisiae* and later, by Inohara-Ochi et al. with the enzyme from *Bacillus* sp. SAM1606 (here, with the simultaneous synthesis of the glucosyl-sucrose isomers: theanderose and 4^F^-α-d-glucosyl-sucrose) [27, 28]. In addition, trehalulose was synthesised from sucrose using the *Pseudomonas mesoacidophyla* trehalulose synthase, while European honeybee α-glucosidases II and III produced theanderose and erlose as the main products from sucrose glucosylation, respectively [26, 37]. Due to all sugars synthesised in this work are naturally found in honey, we do not discard the possibility that native Mg/Mr-αGlu from nectar yeasts, together with the honeybee α-glucosidases, are involved in the final composition of this natural product. Therefore, the recombinant α-glucosidases from *Metschnikowia* species here characterised for the first time, showed the outstanding capability to produce a variety of hetero-GlcOS with α(1→1), α(1→6), α(1→4) and α or β(1→3) bonds, whose large scale purification from natural sources is very unfeasible. The oligosaccharides synthesised by these enzymes are of great biotechnological relevance due to their applications. For instance, isomelezitose, trehalulose, theanderose and erlose are non-cariogenic sugars with suitable sweetness and low caloric values that could be included in food products as dextrose or sucrose substitutes [9, 32]. Moreover, isomelezitose is a potential prebiotic sugar and thus, a substrate for bifidobacteria [44]. Hence, we are currently addressing different immobilization strategies of Mg/Mr-αGlu enzymes for continuous honey hetero-GlcOS overproduction. In addition, the potential application of this sugar mixture as prebiotics as well as the possibility of improving the purity of the hetero-GlcOS mixture by removing glucose and fructose excess are also being evaluated.

Since recombinant Mg/Mr-αGlu displayed negligible hydrolytic activity towards Glc-(1→1)-Glc bonds (Fig. 4), trehalulose production was examined in order to confirm that the synthesis was produced by free fructose glucosylation and not by the formation and rapid hydrolysis of the hypothetical trisaccharide Glc-(1→1)-Fru-(2→1)-Glc. Thus, reactions containing 150, 200 and 250 g/L sucrose plus 150, 100 and 50 g/L free fructose, respectively, were performed in the standard conditions described above. Control reactions without fructose were also followed and all mixtures were analysed by HPLC-ELSD. Figure 5c shows the molar ratio of glucose present in trehalulose or isomelezitose related to the total initial glucose amount. As could be detected in all conditions analysed, the ratio glucose transferred vs. total glucose in isomelezitose synthesis is practically the same comparing the reactions with or without free fructose. As expected, the formation of this trisaccharide depended on the glucosylation of non-hydrolysed sucrose in the reaction medium. However, the molar ratio of glucose transferred *vs.* total glucose in trehalulose production was significantly higher in reactions with fructose added, compared to control reactions. Consequently, the formation of the disaccharide Fru-(1→1)-Glc seemed to occur by glucosylation of released fructose at 1-OH position (Additional file 1: Fig. S4).

### Hetero-GlcOS production from sucrose

**Fig. 6:**
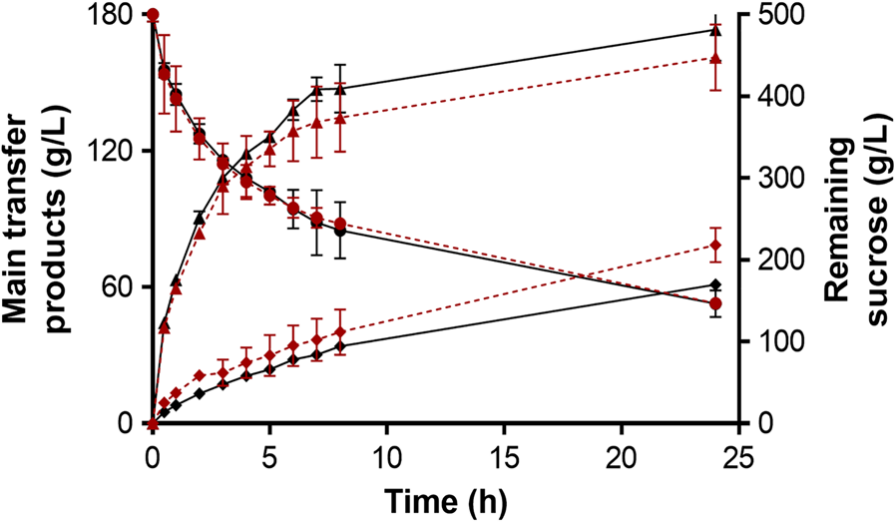
Time course of trehalulose (*rhombus*) and isomelezitose (*triangles*) produced by Mg-αGlu (*red dotted lines*) and Mr-αGlu (*continuous black lines*). Remaining sucrose is indicated (*circles*). Standard errors are indicated

The evolution of hetero-GlcOS formation mediated by Mg/Mr-αGlu evaluated during 24 h showed that proportions of isomelezitose and trehalulose were very similar regardless the recombinant enzyme employed (Fig. 6). After 24 h of reaction progress, the maximum hetero-GlcOS yield was reached when ~ 71% (w/w) of sucrose was consumed. At this point, Mg-αGlu produced ~ 52% (w/w) of hetero-GlcOS (259 g/L, of which 161 g/L corresponded to isomelezitose, 79 g/L to threalulose, and 19 g/L to a mixture of erlose, melezitose, theanderose and erlose). Besides, Mr-αGlu generated ~ 51% (w/w) of hetero-GlcOS (256 g/L, of which 173 g/L corresponded to isomelezitose, 61 g/L to threalulose, and 22 g/L to the rest of transglucosylation products). Here, performing the reactions with an apparent pure enzyme and under the optimal conditions of pH and temperature, isomelezitose production was increased almost twofold compared to our previous work (~ 81 g/L; 120 h reaction; ~ 76% (w/w) of sucrose consumed), where a cell extract from *M. reukaufii* was used [32]. It is worth mentioning that, to our knowledge, heterologous α-glucosidases from *M. gruessii* and *M. reukaufii* described in this work are the mayor producers of isomelezitose, not improved by molecular bioengineering. For instance, employing the partial purified α-glucosidase from *S. cerevisiae* and 100 g/L sucrose, isomelezitose constituted ~ 0.4% of sugars in the reaction mixture [27]. In addition, maximum isomelezitose yield was ~ 8% of total sugar when α-glucosidase from *Bacillus* sp. and sucrose 600 g/L were used, while Munir and Vogel (1999) patented the industrial production of this trisaccharide employing immobilized *Protaminobater rubrum* cells with yields not exceeding ~ 11% at 50 °C [28, 44].

## Conclusions

The search and characterization of new enzymes implicated in bioactive oligosaccharides production have enormous biotechnological interest. In this work, two α-glucosidases from nectaries yeasts of the genus *Metschnikowia* were molecularly characterized and overproduced for the first time. The enzymes were employed in overload sucrose reactions to generate different hetero-GlcOS, which are naturally components of honey. Isomlezitose and trehalulose were the main synthesised products, with the highest isomelezitose yields reported to date. The oligosaccharide mixture included sugars with interesting applications in food and medical fields. Understanding how these enzymes synthesise these sorts of oligosaccharides is fundamental for their biotechnological application and improvement by molecular bioengineering techniques.

## Methods

### Chemicals

Glucose, fructose and sucrose were from Merck (Darmstadt, Germany). Isomaltose, maltose, maltotriose, melezitose monohydrate, palatinose, trehalose, soluble starch, *p*-nitrophenol, *p*-nitrophenol-α-d-glucopyranoside, kanamycin sulfate, 1,4-dithiothreitol and 2,3,5-triphenyltetrazolium chloride were from Sigma-Aldrich (Saint Lois, MO, USA). Yeast extract, peptone, tryptone and agar were from Laboratorios Conda S.A. (Madrid, Spain).

### Strains, grow and expression media

*Metschnikowia gruessii* 2F7.2 and *M. reukaufii* 1LL10 were isolated from *Helleborous foetidus* nectaries (Sierra de Cazorla, southeastern Spain) by Dr. Carlos M. Herrera (Estación Biológica de Doñana (CSIC), Seville, Spain). Yeast were maintained in YEP(D) solid media (10 g/L yeast extract, 20 g/L peptone, 20 g/L glucose and 20 g/L agar) at 4 °C. YEP-liquid media containing 20 g/L glucose (D) or sucrose (S) were used for yeast cultures at 30 °C and orbital shaking (200 rpm). Growth was monitored spectrophotometrically at a wavelength of 660 nm. *Escherichia coli* DH5α and BL21 (DE3), both from Invitrogen (Carlsbad, CA, USA) were used as cloning and expression host, respectively. The recombinant bacteria were maintained in Luria-Bertani (LB) plates (10 g/L peptone, 5 g/L yeast extract, 10 g/L sodium chloride and 20 g/L agar) with kanamycin 30 μg/mL.

### DNA amplification, cloning and site directed mutation

Genomic DNA (gDNA) from *M. gruessii* and *M. reukaufii* was obtained using the procedure described by Polaina and Adam [45], with the modifications introduced by Latorre-Garcia et al. [46]. Basically, yeast were cultured in YEP(D) or (S) in a 25 mL flask with orbital shaking during 16 h. Cells were harvest by centrifugation (13,000 rpm), re-suspended in SDS 3% (w/v) and heated at 55 °C for 15 min. Then, 3 M potassium acetate was added; samples were kept on ice for 10 min and centrifuged at 4 °C as above. DNA was precipitated by adding one volume of cooled isopropanol, recovered by centrifugation and treated with 1.0 mg/mL RNase (ThermoFisher, Waltham, Massachusetts, USA).

A putative oligo-1,6-glucosidase coding sequence (CDS) from *M. reukaufii* MR1 (ID CM010598.1, project accession no. PRJNA336445) was kindly supplied by Dr. Manpreet K. Dhami (Fukami Lab, Standford University, Standford, CA, USA) and used for Mr-αGluF and Mr-αGluR primer design (Table 2), which are directed to positions 1–21 and 1669–1699 of the potential CDS (from the initial ATG to final TAG-stop codons), respectively. Fragments of ~1700 bp were amplified from *M. gruessii* (*mg*-*αGlu*) and *M. reukaufii* (*mr*-*αGlu*) gDNA and purified from agarose gel using the NZYGelpure kit (NzyTech, Lisbon, Portugal). The PCR products were included into the pSTBlue-1 plasmid (Novagen, Darmstadt, Germany) and sequenced (Macrogen Spain, Madrid, Spain). The amplified fragments showed 98.77 and 98.12% identity with the *M. reukaufii* MR1 α-glucosidase CDS, respectively, and were cloned into the pET28b(+) expression vector (Novagen, Darmstadt, Germany) using the restriction-free method reported by Van den Ent and Löwe based in two PCR reactions [47]. Frist, a PCR fragment containing the gene of interest (*mg*-*αGlu* or *mr*-*αGlu*), fused to short sequences complementary to sequences flanking the site of insertion in pET28b(+) were amplified utilizing the primers RFpET28F and RFpET28R (Table 2). New PCR amplicons were purified from agarose gel as before and used as mega-primers in the second amplification reaction using pET28b(+) as template. Phusion High-fidelity DNA polymerase (NEB, Ipswich, UK) was used. Conditions for the first PCR reaction were: (i) 98 °C for 30 s; (ii) 30 cycles of 98 °C for 10 s, 55 °C for 30 s, 72 °C for 60 s; (iii) 72 °C for 420 s, and for the second PCR reaction, the same as the first but with an extension time of 240 s. Then, PCR mixtures were treated with *Dpn*I to digest the methylated parental plasmid and transformed into competent *E. coli* cells by electroporation using standard techniques. Positive clones were selected by colony-PCR using the universal primers T7 Promoter and T7 Terminator, both from Sigma-Aldrich (Saint Lois, MO, USA). Transformants with the empty vector pET28b(+) were also obtained and used as controls. The final constructions MgαGLU-pET28b(+) and MrαGLU-pET28b(+) included a ~1720 bp fragment each, flanked by the T7 lac promoter and a C-terminus 6His-tag followed by the stop codon TGA, that contained the potential α-glucosidase gene of the two *Metschnikowia* species. Thus, the encoded proteins would be fused to a 6 histidine-tag at their C-terminal end.

**Table 2:** Primers used in this study

Primer	Sequence
Mr-αGluF	**ATG**ACTGACGCTATTTGGTGG
Mr-αGluR	**CTA**GTTCTTGACAATGTACAATCTACTCTC
RFpET28F	CCCCTCTAGAAATAATTTTGTTTAACTTTAAGAAGGAGATATACCATGACTGACGCTATTTGGTGGAAAG
RFpET28R	GATCTCAGTGGTGGTGGTGGTGGTGGTTCTTGACAATGTACAATCTACTCTCG
D206AF	TCAGAATCGCGACTGCTGGTATGTACTCCAAGGTGCAAACCTTTGAGG
D206AR	ACCAGCAGTCGCGATTCTGAAACCGTCCACACCCTTCTCGAACCAG
E264QF	ACTGTTGGTCAGGTGGGCCACTCTTCTCGTGAAGATGCCTTGAAGTACGTC
E264QR	GTGGCCCACCTGACCAACAGTCATGGCGTCGTACTTGGAGGTCACCTTC
D339AF	GAGAACCACGCGCAACCAAGATGTATCACTCGTTTCGGTAACGACAGCCCAG
D339AR	CATCTTGGTTGCGCGTGGTTCTCGAAGAAACAAGTTGACCATGCATCAGTTCC

Sequences complementary to the insertion site in the pET28b(+) plasmid are underlined. Initial ATG and TAG stop codons are indicated in bold

Site-directed mutagenesis was carried out according to the protocol described by Liu and Naismith [48] with slightly modifications. Briefly, the constructions MgαGLU-pET28b(+) and MrαGLU-pET28b(+) were amplified in a PCR reaction using pairs of primers containing the desired mutations (D206A, E264Q and D339A). Then, PCR products were treated with *Dpn*I and used to transform *E. coli* cells. All generated constructions were verified by sequencing.

### Expression and purification of the α-glucosidase from *Metschnikowia* species

For protein expression, transformed *E. coli* cells were inoculated on 10 mL of LB media supplemented with 30 μg/mL kanamycin, and cultures were maintained at 30 °C with orbital shaking (200 rpm) during 14–16 h. Then, the culture was used to inoculate 500 mL of LB media including kanamycin and maintained during 4–5 h (final OD_600_ ~ 0.8–1). At this point, protein expression was induced by adding isopropyl-β-d-1-thiogalactopyranoside (IPTG) to a final concentration of 1 mM, followed by incubation at 20 °C for 14–16 h. Cells were precipitated by centrifugation (6700×*g*, 15 min) and resuspended in ~ 40 mL of washing buffer (20 mM sodium phosphate pH 7.5, 200 mM sodium chloride, 20 mM imidazole, 1 mM 1,4-dithiothreitol). Cell suspensions were sonicated using a MSE 150 Watt Ultrasonic Disintegrator Mk2 (MSE Scientific Instruments, Sussex, England), followed by a centrifugation step (14,640×*g*) for 45 min. Non-precipitated cell lysates were filtered through 0.45 μm syringe PVDF filters (Merck Millipore, Burlington, MA, USA). The His-tagged recombinant proteins were easily (but non efficiently; see Additional file 1: Table S1) purified by affinity chromatography using His GraviTrap columns (GE Healthcare Life Science, Buckinghamshire, UK) at 4 °C and recovered with elution buffer (same as the washing buffer, but with 500 mM imidazole). The fractions containing purified protein variants (wild-type or mutated) were dialysed against 20 mM HEPES buffer pH 7.0 at 4 °C for 16–18 h and frozen at − 70 °C.

### Sequence analysis and molecular modelling

Sequences encoding α-glucosidases from *M. gruessii* and *M. reukaufii* have been assigned to the GenBank accession nos. MT161599 and MT161600, respectively (Additional file 1: Fig. S5). Amino acid sequences were analysed using the National Center for Biotechnology Information (NCBI) tool SmartBlast (https://blast.ncbi.nlm.nih.gov/smartblast/). Theoretical molecular weights were determined using ProtParam (https://web.expasy.org/protparam/) on ExPASy. Multiple alignments were carried out with the program Clustal Omega (https://www.genome.jp/tools-bin/clustalw) [Pairwise alignment gap open penalty 10, gap extension penalty 0.1; Multiple alignment gap open penalty 10, gap extension penalty 0.05] and analysed using the application Jalview 2.10.5. Protein modelling, structural predictions and analysis were performed employing Phyre2 [49], the Swiss-model server from ExPasy repository (https://swissmodel.expasy.org/) and the program UCSF Chimera [50]. The sequence logo for the conserved sequence regions (CSR) was performed employing the WebLogo web-based application (http://weblogo.threeplusone.com/).

### SDS-PAGE and zymogram analysis

BlueSafe stained (NzyTech, Lisbon, Portugal)-sodium dodecyl sulphate-polyacrylamide gel electrophoresis (SDS-PAGE; 12%) confirmed their protein level. Precision Plus Protein Standards Unstained 10–250 kDa (Bio-Rad, Hercules, CA, USA) were used as molecular weight markers. Protein concentration was determined using Bio-Rad Protein Assay and bovine serum albumin (Sigma-Aldrich, Saint Lois, MO, USA) as standard, or by measuring absorbance at 280 nm with a NanoDrop ND-1000 spectrophotometer (ThermoFisher, Waltham, Massachusetts, USA).

Hydrolytic activity towards sucrose was detected by zymogram analysis using the methodology described by Linde et al. [51]. Briefly, proteins were resolved on non-denaturing gels (PAGE 12% without SDS) at 4 °C and 120 V. After electrophoresis, gels were washed twice with 50 mM sodium phosphate pH 7.0 containing 1% (v/v) Triton X-100. Then, a solution of 1 M sucrose in the same buffer was added and gels were incubated 1 h at 30 °C. Finally, gels were extensively washed with distilled water and the reducing sugars (glucose and fructose) were revealed using 2,3,5-triphenyltetrazolium chloride (TTC). Commercial *Saccharomyces cerevisiae* invertase (Novozymes, Bagsvaerd, Denmark) was used as control.

### Enzyme and kinetic analysis

Sucrose hydrolytic activity was determined by the dinitrosalicylic acid (DNS) method adapted to a 96-well microplate scale [32]. The reaction mixture (50 μL) containing 60 g/L sucrose in 50 mM sodium phosphate pH 7.0 (45 μL) and the enzyme solution (5 μL) conveniently diluted to fit into the calibration curve (glucose in the 0–3 mg/mL range) was incubated at 30 °C for 15–20 min. The reaction was stopped by adding 50 μL of DNS and then incubated at 80 °C for 30 min. One unit of sucrose hydrolytic activity was defined as that catalysing the formation of 1 μmol of reducing sugar per minute. To compare the specific hydrolytic activity towards different reducing and non-reducing sugars, the glucose released at 30 °C was determined using the GOD-POD colorimetric method kit (NzyTech, Lisbon, Portugal) adapted to a 96-well scale. All substrates were 20 g/L except soluble starch, which was used at a concentration of 10 g/L. A glucose calibration curve (0–1 mg/mL) was used and one unit of hydrolytic activity was defined as the amount of enzyme that liberated 1 μmol of glucose per minute. When using 2 mM *p*-nitrophenol-α-d-glucopyranoside (pNPG) as substrate, a *p*-nitrophenol (pNP) calibration curve (0–0.1 mg/mL) was employed and 1 unit of pNPG hydrolytic activity was defined as that catalysing the formation of 1 μmol of pNP per minute. Reactions without enzyme or substrate were used as negative controls. Each reaction was performed at least in triplicate.

The estimation of the sucrose hydrolytic activity at different pH (4.0–9.5) and temperatures (15–60 °C) values was performed under the aforementioned standard conditions, using sucrose as substrate. The buffers employed were citric acid/sodium citrate (pH 4.0–5.5), Na_2_HPO_4_/NaH_2_PO_4_ (pH 5.5–8.5) and glycine/NaOH (pH 8.5–9.5), all 50 mM. The pH and thermal stability of Mg/Mr-αGlu were determined by pre-incubating 0.02–0.05 U of the apparently pure enzymes for 30–90 min at different pH (4.0–9.0) and temperatures (30–50 °C). Samples were removed at regular intervals and the residual hydrolytic activity was estimated. Enzyme half-life (T_50_) is referred to the temperature needed for 50% of the enzyme inactivation. All reactions were performed in triplicate.

The Michaelis-Menten kinetic constants were determined using 0–225 mM sucrose and 0–180 mM maltose. The plotting and analysis of the curves were carried out using Graph Pad Prism software (version 6.01), and the kinetic parameters were calculated fitting the initial rate values to the Michaelis-Menten equation.

### Gluco-oligosaccharides production

The transferase activity was tested using 500 g/L sucrose in 50 mM sodium phosphate pH 7.0, 30 U of sucrose hydrolytic activity, at 30 °C and 650 rpm in a Vortem 56 shaker (Labnet International, USA). The final reaction volume was 1.0 mL. Samples of 50 μL were withdrawn at different reaction times (0–24 h), maintained at 100 °C for 10 min to inactivate the enzyme, and store at − 20 °C. In order to analyse the trehalulose formation through free fructose glucosylation, reactions containing 150, 200 and 250 g/L sucrose with 150, 100 and 50 g/L fructose, respectively (all in 50 mM sodium phosphate pH 7.0) and 30 U of hydrolytic activity were incubated at 30 °C and 650 rpm. Control reactions without fructose were carried out. Reactions were kept until all sucrose was consumed and then analysed by HPLC-ELSD. For HPLC analysis, samples were conveniently diluted in distilled water and filtered through 0.45 μm pore size filters (Scharlau, S.L.; Sentmenat, Spain). Each reaction was performed in duplicate or triplicate.

### Quantification of GlcOS and hetero-GlcOs by HPLC

GlcOS production was followed by HPLC using a quaternary pump (Delta 600, Waters) coupled to a Kromasil-NH2 column (250 × 4.6 mm, 5 μm) from Análisis Vínicos S.L. (Tomelloso, Spain). Detection was performed using an evaporative light scattering detector (ELSD, mod. 1000, Polymer Laboratories, Ltd.; Church Stretton, UK) equilibrated at 90 °C. Column temperature was kept constant at 30 °C. Analytes were eluted with a mixture acetonitrile/water 80:20 (v/v), degassed with helium, at flow rate of 1.0 mL/min for 30 min. Data obtained were analysed using the Millenium32 Software. Sugars were also analysed by high-performance anion-exchange chromatography with pulsed amperometric detection (HPAEC-PAD) on a Dionex ICS3000 system and a CarboPack PA-1 column (4 × 250 mm) connected to a PA-1 guard column. An electrochemical detector with a gold working electrode and Ag/AgCl as reference electrode was used. The initial mobile phase was 100 mM NaOH for 8 min. Then, a gradient from 100–88% 100 mM NaOH and from 0–12% 100 mM NaOH/600 mM sodium acetate was performed in 22 min. This last mobile phase composition was kept for 6 more minutes and then changed to 50% 100 mM NaOH and 50% 100 mM NaOH/600 mM sodium acetate. The flow rate was 1 mL/min during the analysis. Eluents were degassed by flushing with helium, and peaks were analysed using Chromeleon software. Glucose, fructose, melezitose monohydrate and sucrose were used as standards.

## Supplementary information

**Additional file 1: Table S1.** Purification steps of recombinant Mg/Mr-αGlu. **Figure S1.** Multiple sequence alignment of recombinant Mg-αGlu, Mr-αGlu and glycoside hydrolases from family GH13. The two recombinant α-glucosidases analysed in this work were aligned with the α-glucosidase from *S. pombe* (UniProtKB: Q9P6J3) and the oligo-1,6-glucosidases from *S. cerevisiae* (PDB: 3A47_A) and *B. cereus* (PDB: 1OUK_A). Colour code: identical residues conserved among the proteins are depicted in red while the catalytic triad (Asp206, Glu264, Asp339) are shown in yellow. The four conserved sequence regions (CSR) I-IV characteristic of the family GH13 are boxed. Asterisks mark the conserved amino acids proposed to comprise the binding subsite -1. Secondary structure elements are indicated above the alignment as follows: cyan and green the β-strands and α-helices, respectively, that constitute the catalytic domain A. Dark orange the short β-strands of the domain B and in yellow the seven β-sheets of the C-terminal domain C. **Figure S2.** Analysis of inactive variants of the recombinant proteins. SDS-PAGE of the Mr-αGlu purified fractions (~ 1–3 μg of total protein): wild type (lane 1), D206A mutant (lane 2), E264Q mutant (lane 3), D339A mutant (lane 4) **(a)**. Zymogram analysis using 1M sucrose and TTC: Mr- αGlu wild-type (lane 1), D206A mutant (lane 2), E264Q mutant (lane 3), D339A mutant (lane 4), all at 1–3 μg of total protein/well. As positive control, 0.5–1 μg of *S. cerevisiae* invertase was used (lane 5) **(b)**. Hydrolytic activity assay using sucrose 60 g/L and DNS. Negative controls: reaction without substrate (1) or without wild-type enzyme (2). Positive controls: glucose + DNS (3) and reaction performed with S. cerevisiae invertase (4). Reactions mediated by Mg-αGlu (left column 5–8) and Mr-αGlu (right columns 5–8) variants: wild-type proteins (5), D206A mutants (6), E264Q mutants (7), and D339A mutants (8) **(c)**. **Figure S3.** Initial rate-substrate profile for recombinant Mg/Mr-αGlu. Non-linear curves were obtained fitting the initial rate as function of substrate concentrations, sucrose 0–225 mM **(a)** and maltose 0–180 mM **(b)** to the Michaelis-Menten model. Red curve correspond to recombinant Mg-αGlu and black curve to Mr-αGlu. Experiments were performed in triplicate. Standard errors are indicated. **Figure S4.** Scheme of the transglucosylation process proposed for the reaction of Mg-αGlu or Mr-αGlu on sucrose. The main transglucosylation products depicted in red. **Figure S5.** Coding sequence (CDS) of *mg-αGlu* (a) and *mr-αGlu* (b) genes. Nucleotide sequences with its corresponding translation product are shown. In both cases, ATG initiation (+1 position) and TAG stop (+1699 position) codons are in bold and boxed. The tree acid residues that comprise the catalytic triad (Asp206, Glu264, and Asp339) are circled in yellow, while the four conserved sequence regions of family GH13 are depicted in red. The differences between *mg-αGlu* and *mr-αGlu* sequences are circled in blue. **Table S2.** Bioactive oligosaccharides produced by transglucosylation reactions mediated by glycoside hydrolases from family GH13 cited in this work.

## Data Availability

All data generated or analysed during this study are included in this manuscript.

## Abbreviations

FOS: Fructo-oligosaccharides
GOS: Galacto-oligosaccharides
GlcOS: Gluco-oligosaccharides
COS: Chito-oligosaccharides
XOS: Xilo-oligosaccharides
GH: Glycoside hydrolase
PAGE: Polyacrylamide gel electrophoresis
HPLC: High performance liquid chromatography
ELSD: Evaporative light scattering detector
HPAEC-PAD: High-performance anion-exchange chromatography
p-NP: *p*-nitrophenol
p-NPG: *p*-nitrophenol-α-d-glucopyranoside
