# Supplementary material for: Molecular characterization and heterologous expression of two α-glucosidases from Metschnikowia spp, both producers of honey sugars
Source: Microb Cell Fact. 2020 Jul 11;19:140. doi: 10.1186/s12934-020-01397-y (PMC7353701; doi:10.1186/s12934-020-01397-y)
Supplement: Supplementary file 1 — Additional file 1: Table S1. Purification steps of recombinant Mg/Mr-αGlu. Figure S1. Multiple sequence alignment of recombinant Mg-αGlu, Mr-αGlu and glycoside hydrolases from family GH13. The two recombinant α-glucosidases analysed in this work were aligned with the α-glucosidase from S. pombe (UniProtKB: Q9P6J3) and the oligo-1,6-glucosidases from S. cerevisiae (PDB: 3A47_A) and B. cereus (PDB: 1OUK_A). Colour code: identical residues conserved among the proteins are depicted in red while the catalytic triad (Asp206, Glu264, Asp339) are shown in yellow. The four conserved sequence regions (CSR) I-IV characteristic of the family GH13 are boxed. Asterisks mark the conserved amino acids proposed to comprise the binding subsite -1. Secondary structure elements are indicated above the alignment as follows: cyan and green the β-strands and α-helices, respectively, that constitute the catalytic domain A. Dark orange the short β-strands of the domain B and in yellow the seven β-sheets of the C-terminal domain C. Figure S2. Analysis of inactive variants of the recombinant proteins. SDS-PAGE of the Mr-αGlu purified fractions (~ 1–3 μg of total protein): wild type (lane 1), D206A mutant (lane 2), E264Q mutant (lane 3), D339A mutant (lane 4) (a). Zymogram analysis using 1M sucrose and TTC: Mr- αGlu wild-type (lane 1), D206A mutant (lane 2), E264Q mutant (lane 3), D339A mutant (lane 4), all at 1–3 μg of total protein/well. As positive control, 0.5–1 μg of S. cerevisiae invertase was used (lane 5) (b). Hydrolytic activity assay using sucrose 60 g/L and DNS. Negative controls: reaction without substrate (1) or without wild-type enzyme (2). Positive controls: glucose + DNS (3) and reaction performed with S. cerevisiae invertase (4). Reactions mediated by Mg-αGlu (left column 5–8) and Mr-αGlu (right columns 5–8) variants: wild-type proteins (5), D206A mutants (6), E264Q mutants (7), and D339A mutants (8) (c). Figure S3. Initial rate-substrate profile for recombinant Mg/Mr-αGlu. No [file 12934_2020_1397_MOESM1_ESM.docx]

**Molecular characterization and heterologous expression of two α-glucosidases from *Metschnikowia* spp, both producers of honey sugars**

Martin Garcia-Gonzalez^1^, Marina Minguet-Lobato^1^, Francisco J. Plou^2^, Maria Fernandez-Lobato^1*^

^1^ *Center of Molecular Biology Severo Ochoa (UAM-CSIC), University Autonomous from Madrid, Campus Cantoblanco, 28049 Madrid, Spain.*

^2^ *Institute of Catalysis and Petrochemistry (ICP-CSIC), Campus Cantoblanco, 28049 Madrid, Spain.*

^*^ For correspondance. E-mail [mfernandez@cbm.csic.es](mailto:mfernandez@cbm.csic.es); Tel. +34 911964492; Fax. +34 911964420.

| Table S1 | Purification data of recombinant Mg-αGlu and Mr-αGlu. |
| --- | --- |
| Figure S1 | Multiple sequence alignment of recombinant Mg-αGlu, Mr-αGlu and glycosyl hydrolases from GH13 family. |
| Figure S2 | Analysis of the inactive mutants from the recombinant proteins. |
| Figure S3 | Initial rate-substrate profile for recombinant Mg-αGlu and Mr-αGlu. |
| Figure S4 | Scheme of the transglucosylation process involving Mg-αGlu or Mr-αGlu. |
| Table S2 | Bioactive oligosaccharides produced by transglucosylation reactions mediated by glycoside hydrolases from family GH13 |
| Figure S5 | Coding sequences (CDS) of *mg-αGlu and mr-αGlu* genes. |

**Table S1.** Purification steps of recombinant Mg-αGlu and Mr-αGlu.

| **Mg-αGlu** | | | | | | |  |
| --- | --- | --- | --- | --- | --- | --- | --- |
| **Purification**  **Step** | **Volume (mL)** | **U_T_** | **Total protein (mg)** | **Specific Activity (U/mg)** | **Purification Factor** | **Yield (%)** | |
| **Cell lysate** | 40.0 | 31977 ± 520 | 482 ± 42 | 66 ± 9 | 1.0 | 100.0 | |
| **Ni-NTA** | 5.0 | 4240 ± 681 | 8.2 ± 0.9 | 527 ± 110 | 8.0 | 13.4 | |
|  |  |  |  |  |  |  | |
| **Mr-αGlu** | | | | | | |  |
| **Purification**  **Step** | **Volume (mL)** | **U_T_** | **Total protein (mg)** | **Specific Activity (U/mg)** | **Purification Factor** | **Yield (%)** | |
| **Cell lysate** | 40.0 | 27540 ± 855 | 508 ± 39 | 54 ± 6 | 1.0 | 100.0 | |
| **Ni-NTA** | 5.6 | 4195± 787 | 7.4 ± 0.6 | 566 ± 154 | 11 | 15.2 | |

**
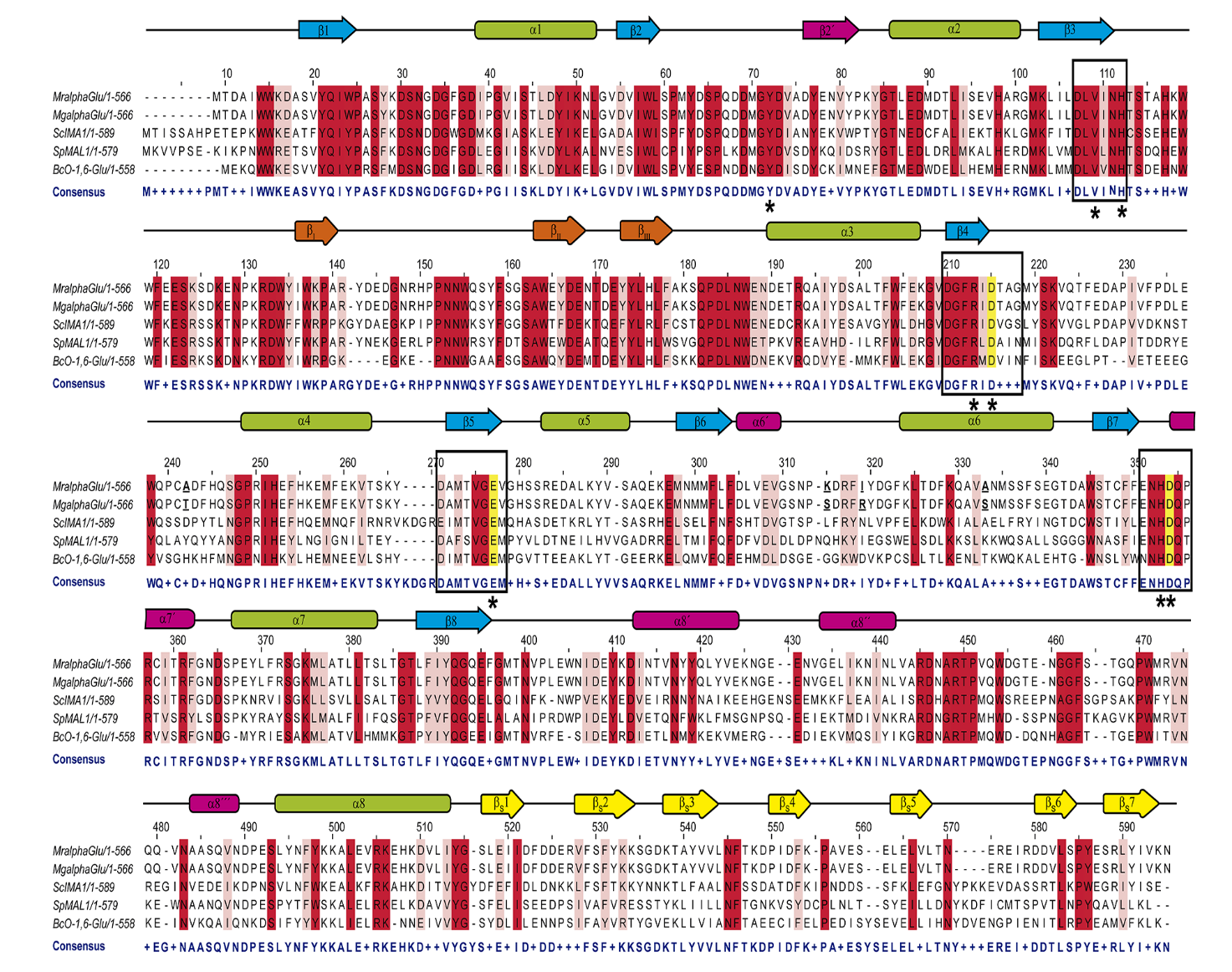
**

**Figure S1.** Multiple sequence alignment of recombinant Mg-αGlu, Mr-αGlu and glycosyl hydrolases from family GH13.

The two recombinant α-glucosidases analysed in this work were aligned with the α-glucosidase from S. pombe (UniProtKB: Q9P6J3) and the oligo-1,6-glucosidases from S. cerevisiae (PDB: 3A47_A) and B. cereus (PDB: 1OUK_A). Colour code: identical residues conserved among the proteins are depicted in red while the catalytic triad (Asp206, Glu264, and Asp339) are shown in yellow. The four conserved sequence regions (CSR) I-IV characteristic of the family GH13 are boxed. Asterisks mark the conserved amino acids proposed to comprise the binding subsite -1. Secondary structure elements are indicated above the alignment as follows: cyan and green the β-strands and α-helices, respectively, that constitute the catalytic domain A. Dark orange the short β-strands of the domain B and in yellow the seven β-sheets of the C-terminal domain C.

**
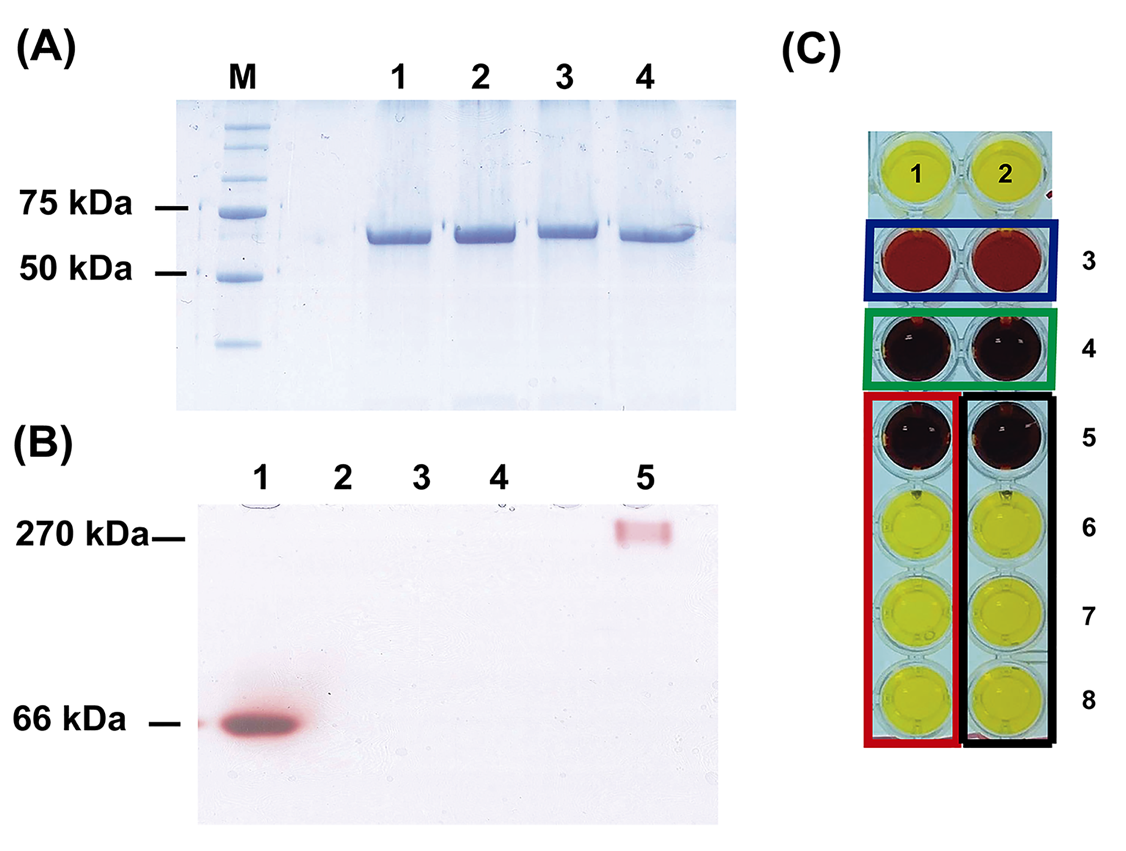
**

**Figure S2.** Analysis of the inactive mutants from the recombinant proteins.

SDS-PAGE of the Mr-αGlu purified fractions (~ 1-3 μg of total protein): wild type (lane 1), D206A mutant (lane 2), E264Q mutant (lane 3), D339A mutant (lane 4) **(a)**. Zymogram analysis using 1M sucrose and TTC: Mr- αGlu wild-type (lane 1), D206A mutant (lane 2), E264Q mutant (lane 3), D339A mutant (lane 4), all at 1-3 μg of total protein/well. As positive control, 0.5-1 μg of *S. cerevisiae* invertase was used (lane 5) **(b)**. Hydrolytic activity assay using sucrose 60 g/L and DNS. Negative controls: reaction without substrate (1) or without wild-type enzyme (2). Positive controls: glucose + DNS (3) and reaction performed with S. cerevisiae invertase (4). Reactions mediated by Mg-αGlu (left column 5-8) and Mr-αGlu (right columns 5-8) variants: wild-type proteins (5), D206A mutants (6), E264Q mutants (7), and D339A mutants (8) **(c)**.


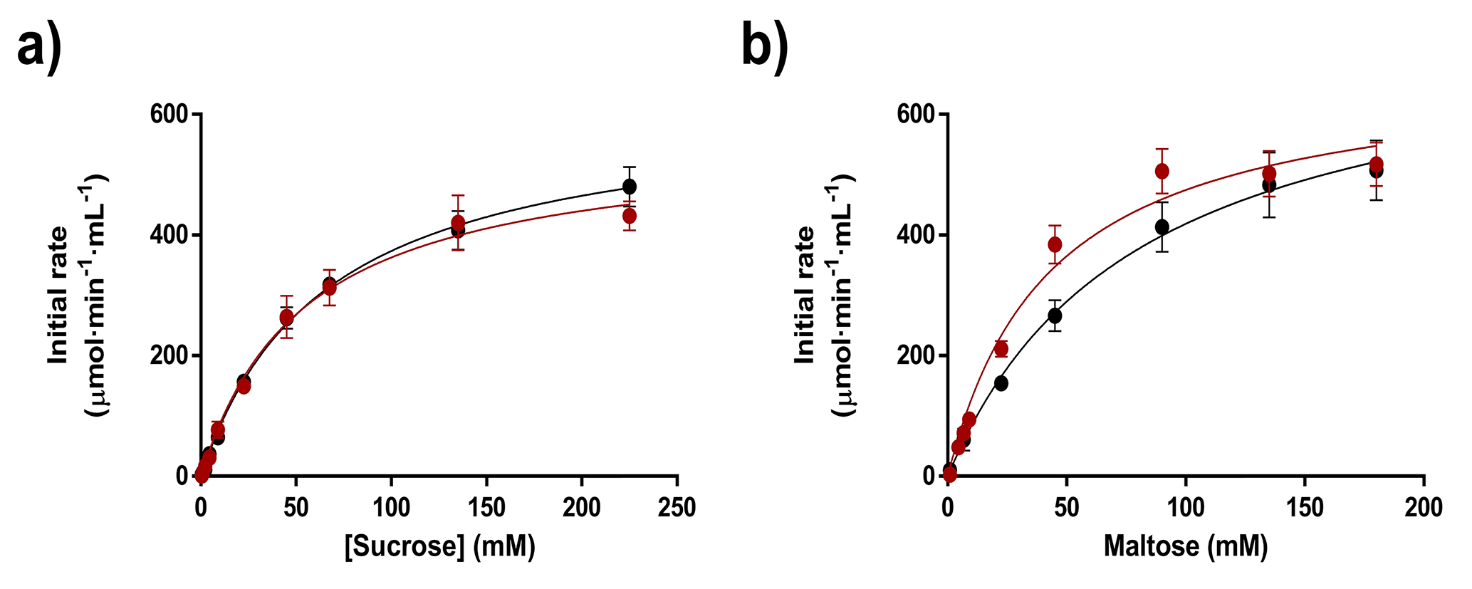


**Figure S3.** Initial rate-substrate profile for recombinant Mg-αGlu and Mr-αGlu.

Non-linear curves were obtained fitting the initial rate as function of substrate concentrations, sucrose 0-225 mM **(a)** and maltose 0-180 mM **(b)** to the Michaelis-Menten model. Red curve correspond to recombinant Mg-αGlu and black curve to Mr-αGlu. Experiments were performed in triplicate. Standard errors are indicated.

**Figure S4.** Scheme of the transglucosylation process proposed for the reaction of Mg-αGlu or Mr-αGlu on sucrose.


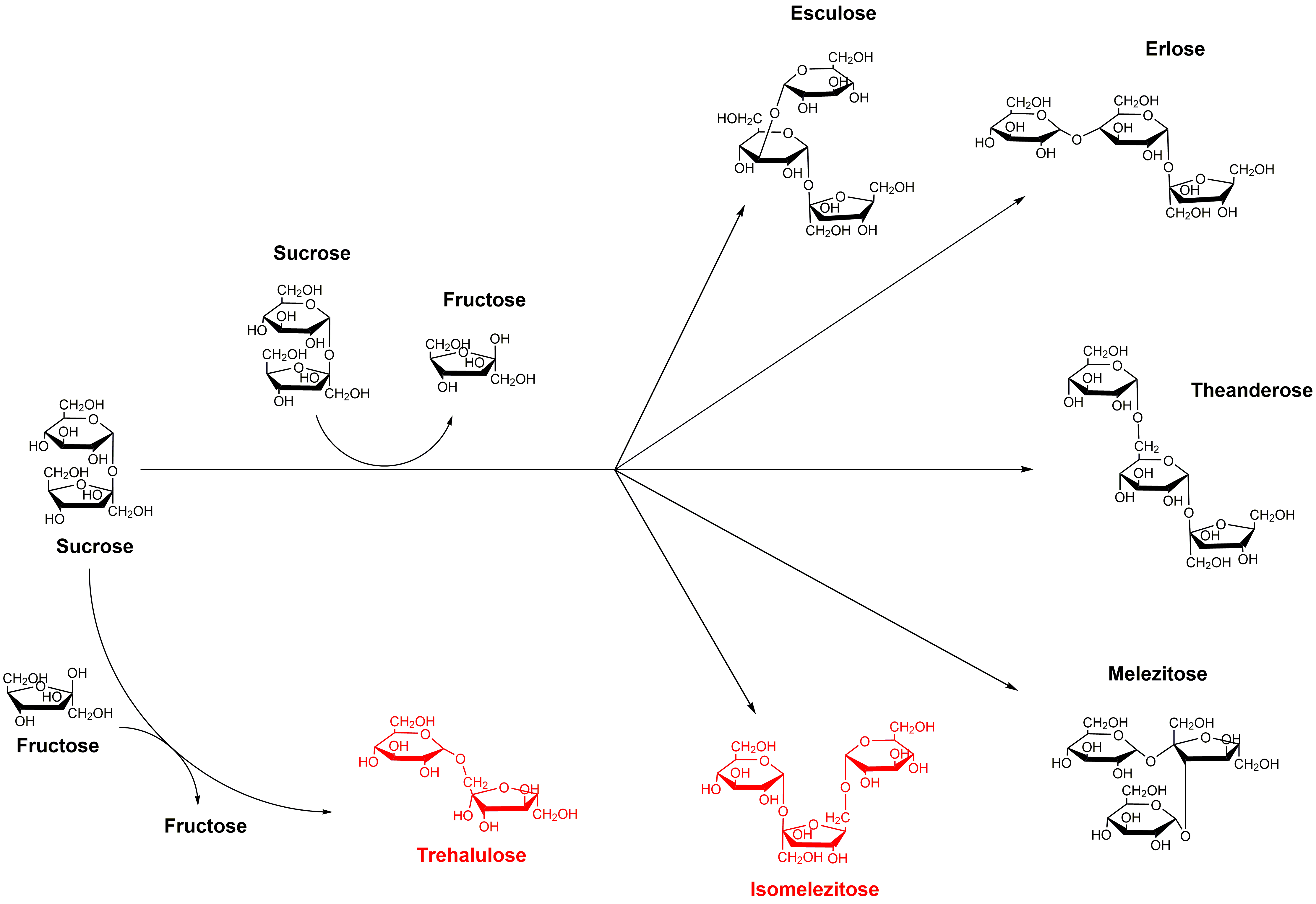


The main transglucosylation products depicted in red.

**Table S2.** Bioactive oligosaccharides produced by transglucosylation reactions mediated by glycoside hydrolases from family GH13 cited in this work.

| **Enzyme** | **Organism** | **Substrate** | **Transfer products (glycosidic bonds formed)** | **Ref.** |
| --- | --- | --- | --- | --- |
| α-glucosidase | *Metschnikowia reukaufii, M. gruessii* | Sucrose | Isomelezitose, trehalulose, theanderose, erlose, melezitose, esculose [α-(1🡪1), α-(1🡪3), α-(1🡪4), α-(1🡪6)] | [32], this work |
| α-glucosidase | *B. licheniformis* | Sucrose | Theanderose  [α-(1🡪6)] | [16] |
| α-glucosidase II and III | *European honeybee* | Sucrose | Theanderose, erlose  [α- (1🡪6), α-(1🡪4)] | [26] |
| α-glucosidase | *Saccharomyces cerevisiae* | Sucrose | Isomelezitose  [α-(1🡪6)] | [27] |
| α-glucosidase | *Bacillus* sp. | Sucrose | Theanderose, isomelezitose  [α-(1🡪6)] | [28] |
| Trehalulose Synthase | *Pseudomonas mesoacidophila* | Sucrose | Trehalulose, isomaltulose  [α-(1🡪1), α-(1🡪6) ] | [37] |

**a)**


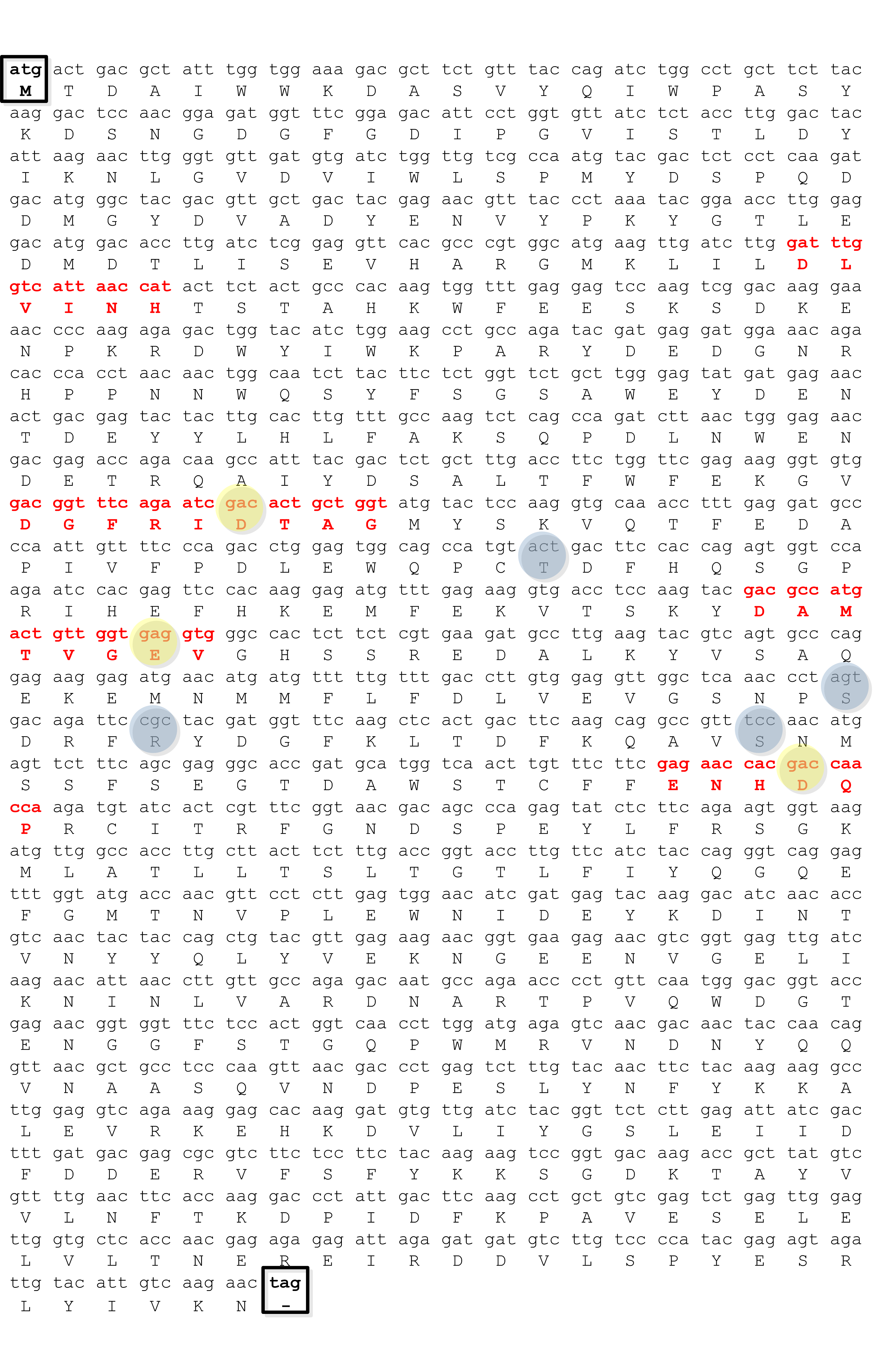


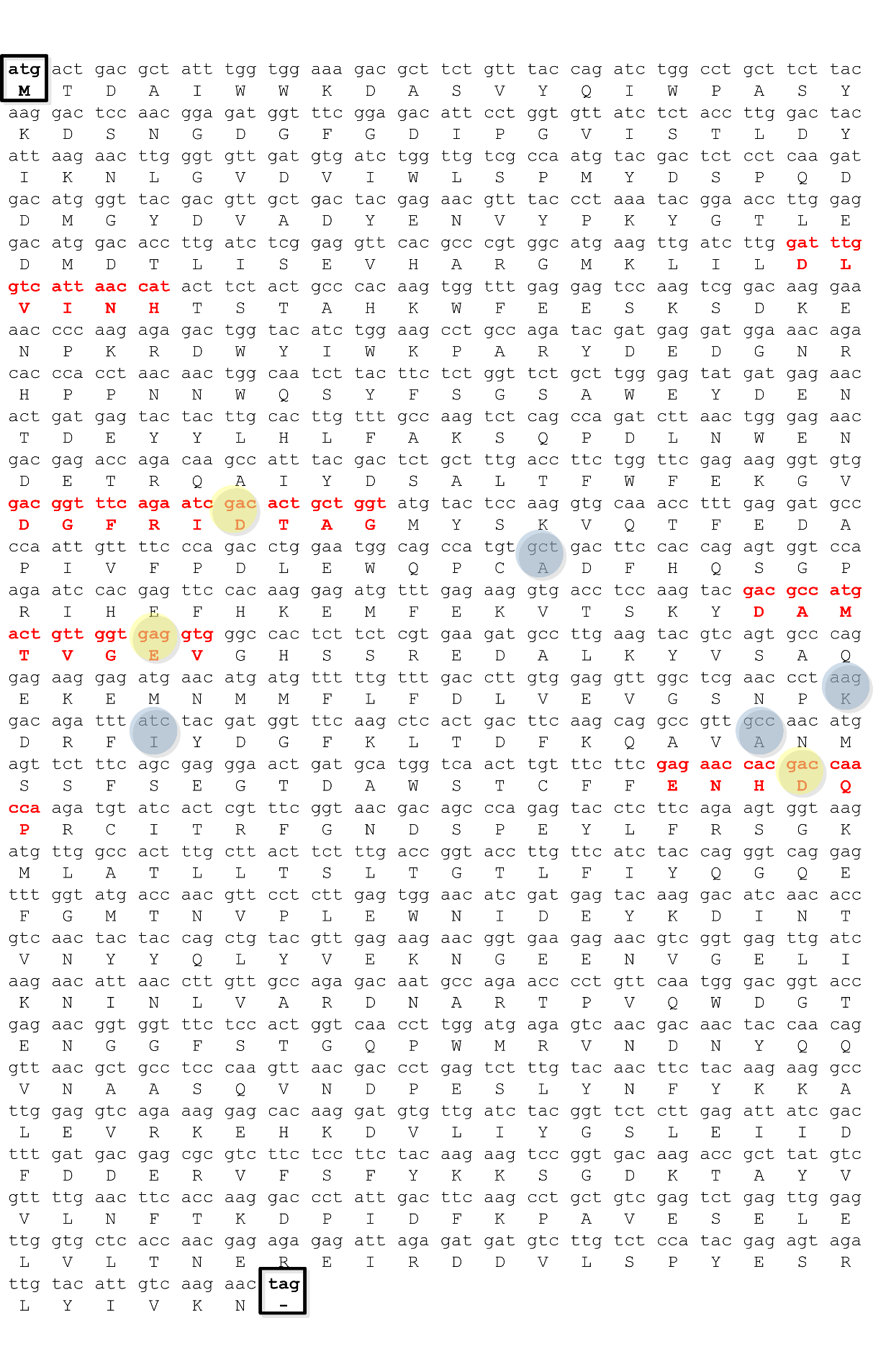


**b)**

**Figure S5.** Coding sequence (CDS) of *mg-αGlu* **(a)** and *mr-αGlu* **(b)** genes.

Nucleotide sequences with its corresponding translation product are shown. In both cases, ATG initiation (+1 position) and TAG stop (+1699 position) codons are in bold and boxed. The tree acid residues that comprise the catalytic triad (Asp206, Glu264, and Asp339) are circled in yellow, while the four conserved sequence regions of family GH13 are depicted in red. The differences between *mg-αGlu* and *mr-αGlu* sequences are circled in blue.
